# Measurement of Lipid Peroxidation Products and Creatine Kinase in Blood Plasma and Saliva of Athletes at Rest and following Exercise

**DOI:** 10.3390/jcm11113098

**Published:** 2022-05-30

**Authors:** Aleksandr N. Ovchinnikov, Antonio Paoli, Vladislav V. Seleznev, Anna V. Deryugina

**Affiliations:** 1 Department of Sports Medicine and Psychology, Lobachevsky University, 603022 Nizhny Novgorod, Russia; 2 Laboratory of Integral Human Health, Lobachevsky University, 603022 Nizhny Novgorod, Russia; antonio.paoli@unipd.it (A.P.); derugina69@yandex.ru (A.V.D.); 3 Department of Biomedical Sciences, University of Padova, 35122 Padova, Italy; 4 Department of Theory and Methodology of Sport Training, Lobachevsky University, 603022 Nizhny Novgorod, Russia; vseleznev92@mail.ru; 5 Department of Physiology and Anatomy, Lobachevsky University, 603022 Nizhny Novgorod, Russia

**Keywords:** saliva, blood plasma, oxidative stress, lipid peroxidation products, muscle damage, creatine kinase, exercise, athletes

## Abstract

This study aimed to assess the agreement between quantitative measurements of plasmatic and salivary biomarkers capable of identifying oxidative stress and muscle damage in athletes at rest and following exercise. Thirty-nine high-level athletes participating in track and field (running), swimming or rowing were recruited and assigned to one of three groups depending on the sport. Each athlete group underwent its specific exercise. Blood and saliva samples were collected before and immediately after the exercise. Diene conjugates (DC), triene conjugates (TC), Schiff bases (SB), and creatine kinase (CK) were measured. Comparisons were made using Wilcoxon signed-rank test. Correlation analysis and Bland–Altman method were applied. DC levels were elevated in plasma (*p* < 0.01) and saliva (*p* < 0.01) in response to exercise in all three groups, as were the plasmatic (*p* < 0.01) and salivary (*p* < 0.01) TC and SB concentrations. CK activity was also significantly higher at postexercise compared to pre-exercise in both plasma (*p* < 0.01) and saliva (*p* < 0.01) in all groups. Strong positive correlation between salivary and plasmatic DC (*p* < 0.001), TC (*p* < 0.001), SB (*p* < 0.01), and CK (*p* < 0.001) was observed at rest and following exercise in each athlete group. The bias calculated for DC, TC, SB, and CK using the Bland–Altman statistics was not significant at both pre-exercise and postexercise in all three groups. The line of equality was within the confidence interval of the mean difference. All of the data points lay within the respective agreement limits. Salivary concentrations of DC, TC, SB, and CK are able to reliably reflect their plasma levels.

## 1. Introduction

Accurate monitoring of oxidative stress and muscle damage caused by exercise is essential in sports and exercise medicine [1]. Recently, the use of saliva has been carefully considered and examined as an attractive option for the assessment of oxidative stress and muscle damage in exercise science due to its non-invasive nature and analyte availability compared to blood analysis [2,3,4,5,6,7,8,9,10,11,12,13,14,15]. Saliva availability allows for the collection of multiple specimens, including continuous supply during each training session, which is not possible with blood sampling [1]. These beneficial aspects of saliva make possible the early recognition of excessive adverse effects induced by exercise.

Additional advantage of saliva over blood collection can be attributed to the simplicity of collection device. Blood sampling requires sample tubes containing clot-activating factors, anti-coagulating and ligand-binding compounds to safely collect and stabilize blood components, while saliva collection simply involves a sterilized tube [4]. Importantly, saliva sampling allows for reduced risk of cross-contamination and personalized timing of sample collection, which does not require a phlebotomist for obtaining a sample [1,14]. Furthermore, blood collection is associated with pain, which may affect secretion of stress hormones. This becomes especially important in studies quantifying oxidative stress following exercise that may lead to false-positive results and overestimation of exercise-induced disruption in redox control.

Concentrations of salivary biomarkers such as cortisol, testosterone, alpha-amylase, glucose, lactate, uric acid, and secretory immunoglobulins have been reported to reliably reflect serum levels [2,4,16,17,18,19,20,21,22]. Despite these advances, salivary biomarkers that are capable of providing accurate information regarding exercise-induced oxidant stress and muscle damage remain to be established. Containing a variety of enzymes, hormones, proteins and lipids, saliva is a potentially ideal medium for the assessment of oxidative stress and muscle damage imposed by the exercise. The most commonly used salivary biomarkers that relate to oxidant stress are thiobarbituric acid reactive substances (TBARS), in particular malondialdehyde (MDA) [1,11,12]. MDA, which is well known, is an intermediate product of lipid peroxidation, and its level cannot give an accurate estimation of the lipid peroxidation process. In addition, only certain lipid peroxidation products generate MDA, and MDA is neither the sole end product of fatty peroxide formation and decomposition, nor a substance generated exclusively through lipid peroxidation. Therefore, the use of MDA analysis and/or the TBA test, as well as interpretation of sample MDA content and TBA test response in studies of lipid peroxidation, require caution, discretion, and correlative data from other indices of fatty peroxide formation and decomposition, especially in biological systems [23]. However, the above-mentioned limitations to use MDA do not apply to some other products of lipid peroxidation such as diene conjugates (DC), triene conjugates (TC) and Schiff bases (SB), which together may provide a reliable indication of oxidative stress. DC are one of the main primary products of lipid peroxidation that refer to two double bonds separated by a single bond [24,25]. TC, in turn, are also alkenes but with three double bonds separated by a single bond in a molecule that can be formed as secondary products during lipid peroxidation [25]. Despite their relative stability compared with free radicals, the chemical structure of DC and TC makes these electrophilic molecules highly reactive [26]. Thus, to better understand the lipid peroxidation rates, it is necessary to also measure the end products of lipid peroxidation with a relatively high stability. The major end product that is derived from the oxidation of arachidonic acid and larger polyunsaturated fatty acids through enzymatic or nonenzymatic processes is α, β-unsaturated aldehyde 4-hydroxynonenal (4-HNE) [26,27]. However, 4-HNE has an extraordinary reactivity that relies upon both the Michael addition of thiol or amino compounds on the C3 of the C2=C3 double bond and the SB formation between the C1 carbonyl group and primary amines [27]. Despite slow and reversible kinetics of SB formation, SB are relatively more stable products than their direct precursor. The advantage over Michael-adducts, and the biggest, is that SB as well as DC and TC are able to be detected by means of simple UV spectrophotometry in both blood plasma and saliva, including under exercise conditions [28]. Collectively, it could make DC, TC and SB predominant to quantify exercise-induced lipid peroxidation and thus oxidative stress.

There is growing evidence that excessive accumulation of toxic lipid peroxidation products during exercise can lead to damage of cellular components in damaged muscle after exercise, what is reflected by increased plasma and saliva levels of soluble muscle enzymes such as creatine kinase (CK) [28,29,30,31]. Consequently, elevated CK activity in saliva following exercise may also be considered as a sign of exercise-induced muscle damage.

There is no study that has evaluated the agreement between quantitative measurements of plasmatic and salivary biomarkers capable of identifying oxidant stress and muscle damage in athletes under exercise conditions. Our aim was to assess the agreement between quantitative measurements of plasmatic and salivary DC, TC, SB, and CK at rest and following exercise. Since exercise has been reported to induce similar pattern of antioxidant response in both blood plasma and saliva [13], as well as the corresponding changes in plasmatic and salivary CK activities [32,33], we hypothesize that salivary DC, TC, SB, and CK could accurately represent plasma concentrations in athletes participating in different sports under special exercise conditions.

## 2. Materials and Methods

### 2.1. Subjects

Thirty nine high-level athletes (sex: Male; age: 19.6 ± 1.1 years; height: 173.3 ± 2.5 cm; body mass: 63.7 ± 2.1 kg; body mass index: 21.2 ± 0.6 kg/m^2^), i.e., athletes who bear qualification in accordance with the Unified Russian Sports Classification System: winners and/or prize-winners of national sports events and international sports events, were recruited from the Olympic Reserve Center (Nizhny Novgorod, Russia) through advertising directly to coaches. Exclusion criteria for all subjects were chronic use of any medication, ingestion of antioxidant supplements, periodontal disease, respiratory infection, and orthopedic injury (sprain, contusion, or fracture within 14 days of enrollment). Each subject participated in one of the three kinds of sports (track and field (running), swimming, rowing) for at least 5 years and thus was assigned to one of three groups depending on the sport. Anthropometric baseline characteristics of subjects are shown in Table 1.

Before any procedures, informed written consent was obtained from each of the participants. The study was conducted in accordance with the Declaration of Helsinki and was approved by the Bioethics Committee of Lobachevsky University (approval number: 43) [34].

### 2.2. Study Design

After signing the informed consent and two weeks before exercise testing, the participants were invited to refer to the Integral Human Health Laboratory of the Faculty of Physical Education and Sport of Lobachevsky University. During the visit, the athletes underwent medical screening and food interview to ensure eligibility for the study and to gather information on the subjects’ dietary habits. Participants were instructed to maintain their usual diet two weeks before the exercise testing. Subjects were also instructed to have breakfast one hour before the training session and refrain from consuming alcohol and caffeinated beverages for at least 24 h before exercise testing. Adherence to study medications was assessed by a qualified dietician with a face-to-face interview.

Each athlete group kept a typical training routine, which was the same for all participants depending on the sport. The group of swimmers comprised short-distance athletes (100 m). Swimmers underwent high-intensity interval exercise (HIIE), which consisted of 4 sets of 50 m distance in their preferred swimming style at the top-most speed interspersed with 45 s of recovery periods in a 25 m swimming pool. The group of runners included only sprinters (100–200 m). Runners underwent their specific HIIE on a 400 m outdoor track of stadium. HIIE protocol applied to the runners consisted of 3 repetitions of 100 m distance at the top-most speed interspersed with 45 s of recovery time. The group of rowers comprised middle-distance rowers specialized in 2000 m. High-intensity continuous exercise (HICE), consisting of 2000 m distance, was performed by rowers at the top-most speed using a rowing ergometer (Concept2, Morristown, VT, USA). Each exercise protocol was selected due to its widespread use in the training process among categories of athletes participated in the study. Exercise testing was performed in the morning. Subjects were examined before and immediately after exercise for blood and saliva sample collection, followed by measurements of oxidative stress and muscle damage biomarkers.

### 2.3. Measurement of Exercise Performance

Runners underwent HIIE consisting of 3 sets of 100 m distance with 45 s rest between the sets. The aim of the test was to complete each set in the shortest possible time. Results were determined by the time taken to complete each set with the subsequent calculation of mean. Timing was recorded using a stopwatch from the start signal until the runner crossed the finish line.

Swimmers underwent HIIE consisting of 4 sets of 50 m distance with 45 s rest between the sets. The aim of the test was to complete each set in the shortest possible time. Results were determined by the time taken to complete each set with the subsequent calculation of mean. Timing was recorded using a stopwatch from the start signal until the swimmer touched the wall to finish. Due to the lack of uniformity requirement for swimming style, in order to unify the results of swimmers, average time to overcome a distance of 50 m by the preferred stroke was converted into the corresponding number of points according to a single assessment system developed and approved by the International Swimming Federation (FINA points).

Rowers underwent HICE consisting of 2000 m distance. The aim of the test was to cover the distance in the shortest possible time. Results were determined by the time taken to overcome the distance. Timing was recorded using a stopwatch from the start signal until the rower overcame the 2000 m.

### 2.4. Sample Collection

Blood from the median cubital vein (approximately 4 mL per collection) was collected and placed in EDTA-coated tubes by a qualified phlebotomist using standardized venipuncture techniques. Saliva samples were collected into the plastic conical centrifuge tubes by a spitting method without stimulation for 3 min. Athletes rinsed their mouth with water before saliva sampling. Subjects were also instructed to swallow the remaining water in the oral cavity and to wait a minute before saliva collection. Blood and saliva samples were taken no more than 5 min before exercise (at rest) and immediately after the exercise. All collection procedures were performed in the morning. Blood and saliva samples were centrifuged at 3000 rpm for 15 min, and supernatant was aliquoted. All samples were stored at −40 °C until analysis.

### 2.5. Measurement of Lipid Peroxidation Products

DC, TC and SB were photometrically determined using spectrophotometer (SF-2000, Saint-Petersburg, Russian Federation). To obtain lipid extract, 0.5 mL of sample (plasma or saliva) was added to 8 mL of heptane–isopropanol mixture in a 1:1 ratio. Then, the sample and heptane–isopropanol mixture were stirred for 15 min and centrifuged at 3000 rpm for 15 min. Subsequently, 5 mL of heptane–isopropanol mixture was added to the lipid extract in a 3:7 ratio. To separate phases and remove non-lipid impurities, 2 mL of aqueous solution of HCl (0.01 N) was added. After phase separation, an upper (heptane) phase was transferred into a clean test tube. To dehydrate the isopropanol extract, 1 g of NaCl was added to a lower phase. Thereafter, the lower phase was transferred to a clean tube. Optical densities (E) were measured at the following wavelengths: 220 nm (absorption of isolated double bonds), 232 nm (absorption of DC), 278 nm (absorption of TC), and 400 nm (absorption of SB). Each phase was assessed against the corresponding control sample, which was prepared in the same way as the test, but distilled water was added instead of plasma or saliva. Sample concentrations of DC, TC and SB are presented as continuous variables and were calculated using standard equations (E232/E220 for calculating DC, E278/E220 for calculating TC, and E400/E220 for calculating SB, respectively) [3,25,28,31,35].

### 2.6. Measurement of Creatine Kinase Activity

CK activity was measured by enzymatic kinetic assay in the range of 1–1100 U/L on biochemical analyzer (Clima MC-15, RAL, Barcelona, Spain) using a CK-NAC DiaS reagent set (Hannover, Germany). When preparing a monoreagent, preheated (37 °C) reagent 1 and reagent 2 were mixed in a 4:1 ratio, respectively. Subsequently, 50 μL of sample (plasma or saliva) was added into a cuvette intended for the sample. Then, 500 μL of monoreagent was added into a cuvette intended for the reagent. Control cuvette was left blank, without reagents and sample. Preheated (37 °C) monoreagent and sample (plasma or saliva) were mixed in a 10:1 ratio. Enzyme activity was measured at 340 nm [3,28,31,33,35].

### 2.7. Statistical Analysis

Descriptive statistical analysis was carried out for all study variables. Data are presented as median and interquartile range (Me, 25th percentile and 75th percentile). Assumption of normality was verified using the Shapiro–Wilk test. Since not all data were normally distributed, comparisons were made using Wilcoxon signed-rank test for all study variables. Statistical relationships between plasmatic and salivary biomarkers at pre-exercise and postexercise were evaluated using Spearman’s correlation coefficient (r_s_). After ensuring that the differences between paired measurements of plasmatic and salivary biomarkers are normally distributed, Bland–Altman plot analysis was applied to assess the agreement between quantitative measurements of the same biomarkers in blood plasma and saliva. For all analyses, a *p* value < 0.05 was considered statistically significant. All statistical analyses were performed using RStudio, version 1.3.1093 for macOS (RStudio, PBC).

## 3. Results

All the recruited subjects successfully completed the study. Exercise performance of subjects is shown in Table 2.

**Figure 1:**
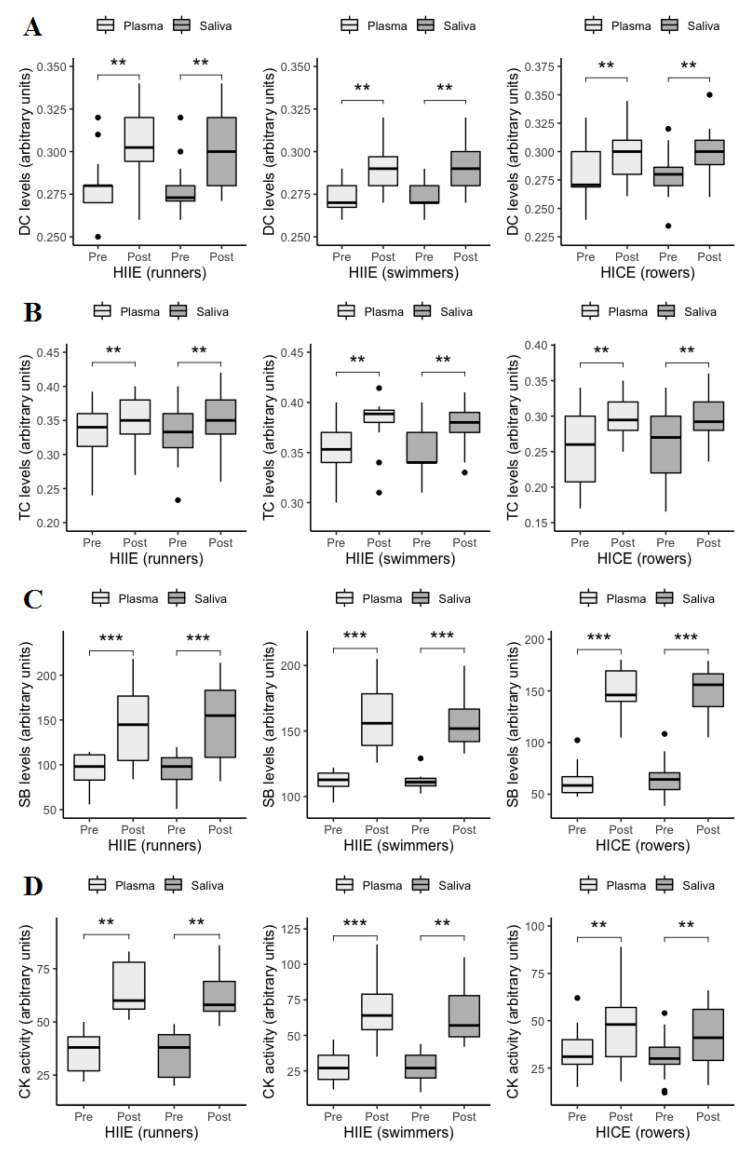
Plasmatic and salivary levels of DC (**A**), TC (**B**), SB (**C**), and CK (**D**) at pre-exercise and postexercise. Data are given as median and interquartile range and compared by Wilcoxon signed-rank test. *n* = 13 per group. ** *p* < 0.01. *** *p* < 0.001. DC: diene conjugates; TC: triene conjugates; SB: Schiff bases; CK: creatine kinase; HIIE: high-intensity interval exercise; HICE: high-intensity continuous exercise.

Measurements of DC, TC, SB, and CK concentrations in both blood plasma and saliva of athletes at rest and following exercise are presented in Figure 1.

The content of plasmatic and salivary DC, TC and SB significantly increased at postexercise compared to pre-exercise in all three groups. Plasmatic and salivary CK activities were also significantly elevated in response to exercise in each group of athletes.

**Figure 2:**
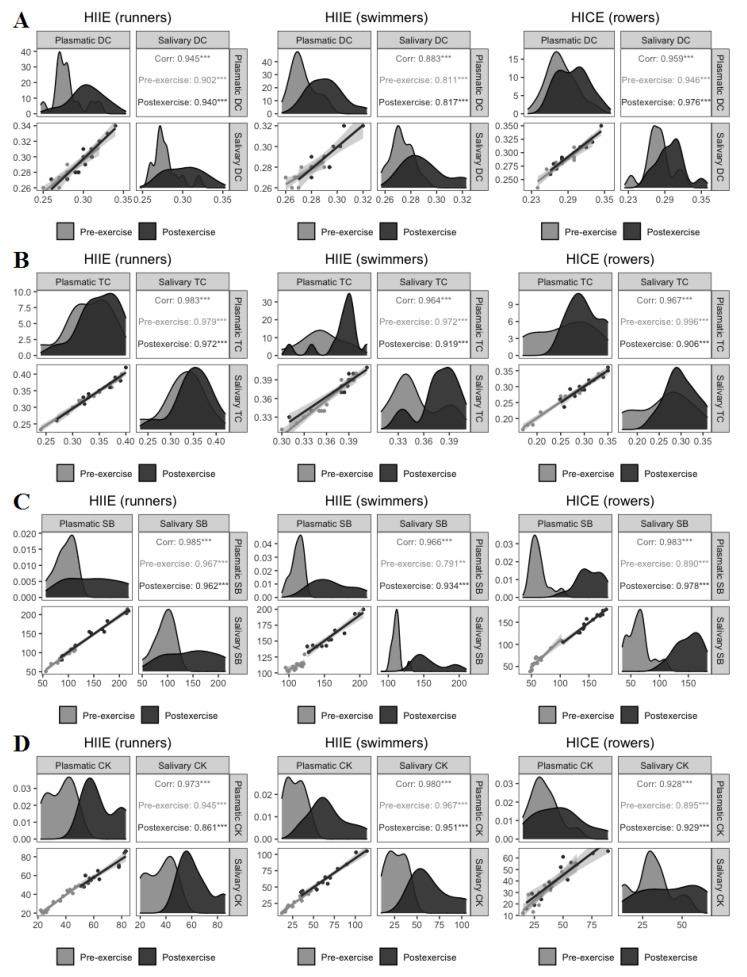
Generalized pairs plots, including density plot, scatter plot and correlation matrix, for displaying distribution and relationship between plasmatic and salivary concentrations of DC (**A**), TC (**B**), SB (**C**), and CK (**D**). *n* = 13 per group. ** *p* < 0.01. *** *p* < 0.001. Corr: Spearman’s correlation coefficient for both pre-exercise and postexercise; Pre-exercise: Spearman’s correlation coefficient at pre-exercise; Postexercise: Spearman’s correlation coefficient at postexercise; DC: diene conjugates; TC: triene conjugates; SB: Schiff bases; CK: creatine kinase; HIIE: high-intensity interval exercise; HICE: high-intensity continuous exercise.

Correlation between plasmatic and salivary concentrations of DC, TC, SB, and CK was evaluated at both pre-exercise and postexercise (Figure 2).

A strong positive correlation between salivary and plasmatic DC, TC and SB levels was observed at rest and following exercise in all groups. It was also verified to have a high positive correlation between plasmatic and salivary CK activities at both pre-exercise and postexercise in each athlete group.

**Figure 3:**
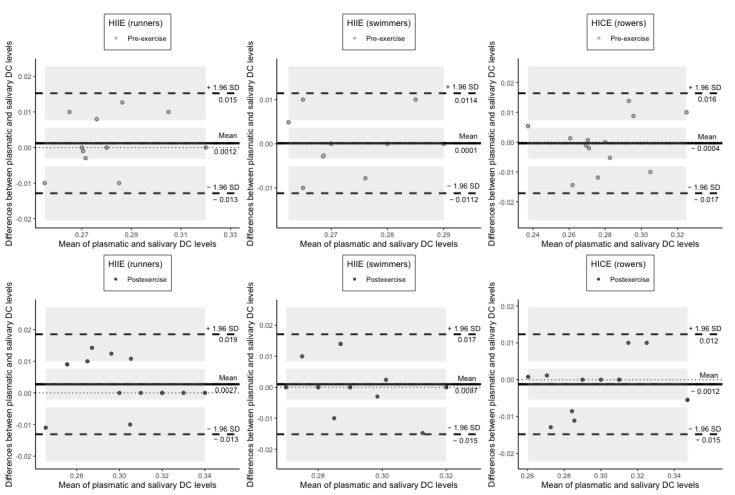
Plots of difference between the measurements of DC levels in plasma and saliva vs. the mean of the measurement. The bias (mean difference) is represented by a solid line parallel to the *X* axis. The limits of agreement are represented by the dashed lines parallel to the *X* axis at −1.96 and +1.96 SD. Shaded areas present confidence interval limits for mean and agreement limits. DC: diene conjugates; HIIE: high-intensity interval exercise; HICE: high-intensity continuous exercise; SD: standard deviation.

Bland and Altman plots allowed us to demonstrate the difference between quantitative measurements of DC, TC, SB, and CK in blood plasma and saliva against the average of the measurements in two biological fluids. In relation to plasmatic and salivary DC concentrations at rest and following exercise, the bias was not significant in all groups because the line of equality was within the confidence interval of the mean difference. At the same time, all of the data points lay within ± 1.96 SD of the mean difference (Figure 3).

**Figure 4:**
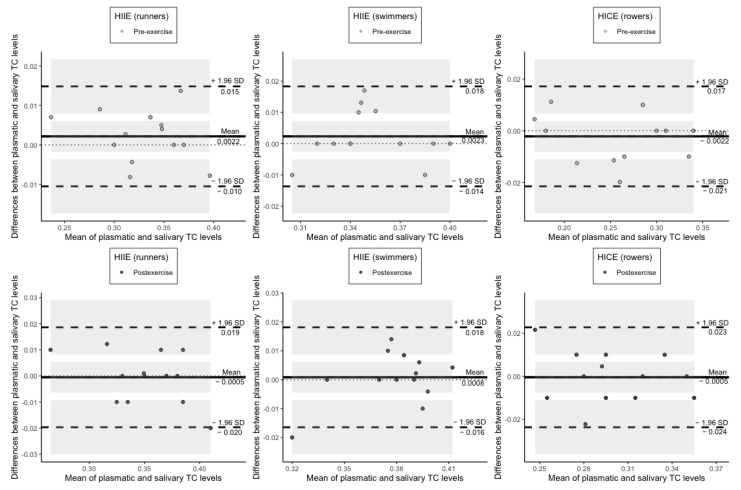
Plots of difference between the measurements of TC levels in plasma and saliva vs. the mean of the measurement. The bias (mean difference) is represented by a solid line parallel to the *X* axis. The limits of agreement are represented by the dashed lines parallel to the *X* axis at −1.96 and +1.96 SD. Shaded areas present confidence interval limits for mean and agreement limits. TC: triene conjugates; HIIE: high-intensity interval exercise; HICE: high-intensity continuous exercise; SD: standard deviation.

The average difference between plasmatic and salivary TC content both at pre-exercise and at postexercise was also not significant in each group of athletes (Figure 4).

**Figure 5:**
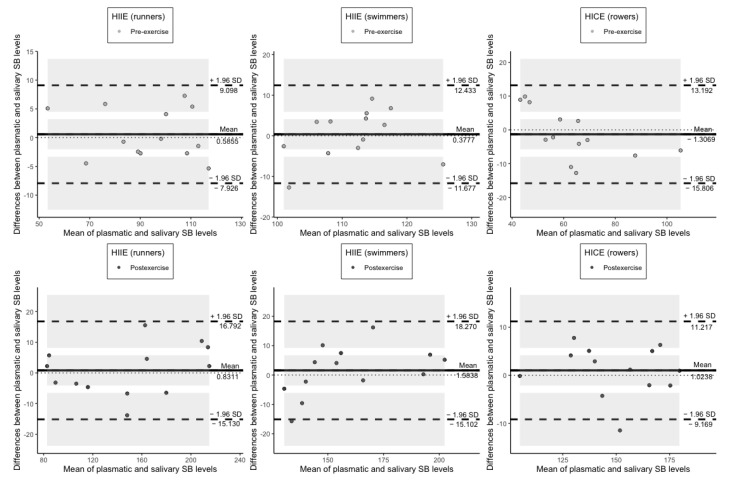
Plots of difference between the measurements of SB levels in plasma and saliva vs. the mean of the measurement. The bias (mean difference) is represented by a solid line parallel to the *X* axis. The limits of agreement are represented by the dashed lines parallel to the *X* axis at −1.96 and +1.96 SD. Shaded areas present confidence interval limits for mean and agreement limits. SB: Schiff bases; HIIE: high-intensity interval exercise; HICE: high-intensity continuous exercise; SD: standard deviation.

Regarding SB levels in plasma and saliva, they were also shown to have a line of equality within the confidence interval of the mean difference both before and immediately after exercise in all three groups (Figure 5).

**Figure 6:**
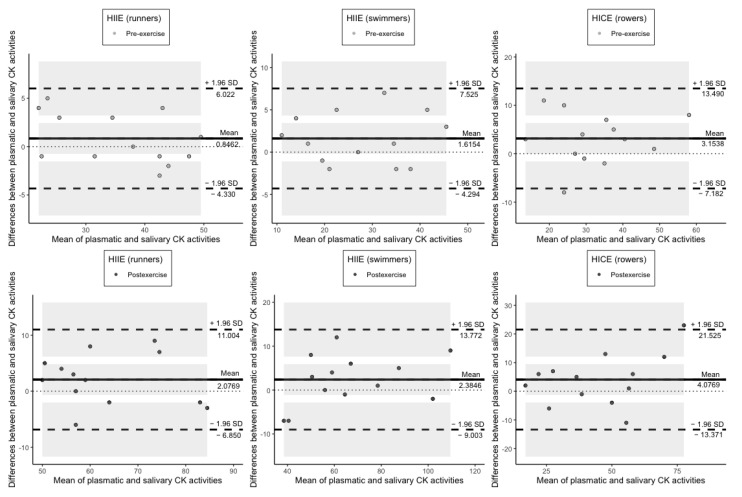
Plots of difference between the measurements of CK activity in plasma and saliva vs. the mean of the measurement. The bias (mean difference) is represented by a solid line parallel to the *X* axis. The limits of agreement are represented by the dashed lines parallel to the *X* axis at −1.96 and +1.96 SD. Shaded areas present confidence interval limits for mean and agreement limits. CK: creatine kinase; HIIE: high-intensity interval exercise; HICE: high-intensity continuous exercise; SD: standard deviation.

As for the measurement of plasmatic and salivary CK activities at rest and following exercise, the bias was not significant in each group of athletes since the line of equality was in the confidence interval (Figure 6).

Simultaneously, all of the data points lay within the agreement limits.

## 4. Discussion

To the best of our knowledge, this is the first study to evaluate the agreement between quantitative measurements of plasmatic and salivary DC, TC, SB, and CK in athletes under exercise conditions. Our results demonstrated that HIIEs associated with running and swimming and HICE related to rowing induce an increase in the content of lipid peroxidation products in both blood plasma and saliva. Although HIIE generally implies less oxygen consumption (due to shorter efforts) than HICE, it could be responsible for significant production of reactive oxygen species (ROS) capable of initiating lipid peroxidation in the active skeletal muscles [36]. Recent data reveal that the rate of electron leakage in the electron transport chain during contractile activity is low enough, enabling only 0.15% of total oxygen consumption to be converted to superoxide radical [30,37,38]. Concurrently, other metabolic pathways such as activation of nicotinamide adenine dinucleotide phosphate oxidase, xanthine oxidase, monoamine oxidase, lipoxygenases are mainly implicated in ROS generation in contracting muscle [38,39]. Exercise-induced changes in salivary DC, TC and SB were shown to be similar to those found in plasma in each athlete group. Moreover, DC, TC and SB levels in saliva reliably reflected the concentrations of these compounds in blood plasma at both pre-exercise and postexercise in all three groups. Similar levels of plasmatic and salivary DC, TC and SB at rest and following exercise may be associated with the fact that these compounds are able to diffuse from serum through capillaries, accumulating in saliva according to the concentration gradient [40]. Our previous work partially confirms these findings where specific HIIEs performed by swimmers and runners induced lipid peroxidation propagation in saliva, which was identified by the measurements of salivary DC, TC and SB levels [3]. However, unlike the present study, there was no blood sampling together with saliva collection (i.e., simultaneously) before and immediately after the exercise, which did not allow us to assess the agreement between quantitative measurements of DC, TC and SB in plasma and saliva.

In addition, it was found that HIIEs for runners and swimmers and HICE for rowers cause increased levels of plasmatic and salivary CK. Importantly, salivary CK activity was consistent with plasmatic CK activity at both pre-exercise and postexercise in each athlete group. These results correspond with our preliminary findings and suggest that salivary CK activity may represent plasma levels of this enzyme at rest and in response to exercise [33]. In a recent study from Barranco et al. [32], the authors also found an increase in CK levels in both plasma and saliva following exercise consisting of a futsal match. More recently, Fernández et al. [41] observed the elevated CK activity in saliva after serial matches of rugby seven.

Increased CK levels found in blood indicate surface membrane damage and disruption of the sarcomere architecture, which may be mediated in part by oxidative stress [29,42]. As is well known, muscle contraction requires a transient increase in intracellular calcium, which is released from the sarcoplasmic reticulum into the cytosol through the excitation–contraction coupling system [29]. An overload of intracellular calcium activates calcium-dependent protease such as calpain-1, which destroys sarcomeric proteins [43]. Although there is no evidence for direct activation of calpain-1 by oxidative stress, the calcium-dependent protease activity is thought to be induced by ischemia/reperfusion damage during exercise due to calcium overload [29,44]. Another mechanism by which oxidant stress-mediated increases in protease activity can occur is activation/sensitization of the ryanodine receptor Ca^2+^-release channels [45]. Current evidence suggests that calpain-1-induced proteolysis of sarcomere proteins is an early process in myocyte injury, and that excess levels of ROS may play a meaningful role in the initiation of signaling events related to muscle damage [29]. Cell membrane modifications triggered by lipid peroxidation propagation often precede irreversible biomolecular damage, being an early cause of cell death [30,46,47]. High concentrations of plasmatic and salivary SB (also known as 4-HNO-protein adducts) were observed in the present study, suggesting that large amounts of 4-HNE were produced during exercise. Lipid peroxidation end-products are able to affect membrane proteins, causing function impairment, increased nonspecific permeability to ions, fluidity changes and inactivation of membrane-bound receptors and enzymes [48,49,50]. Therefore, increased CK levels after exercise may be directly related to lipid peroxidation propagation during exercise [30]. In turn, elevated activity of salivary CK following exercise may be associated with diffusion of this enzyme from the bloodstream via transcellular/paracellular pathways [40,51].

This study has some limitations that should be noted. The first limitation derives from the origin of the sample. The fact that all athletes participated in sprinting, short-distance swimming or middle-distance rowing prompts us to be cautious in generalizing the results to athletes who represent other events and sports. In addition, the results of the present study cannot be extrapolated to the general population, taking into account that the subjects were only athletes. We consider that our research can serve as a basis for further studies enrolling a wider range of participants in order to confirm these findings. Second, it has been stated that CK activity is influenced by gender. Therefore, our results may be conditioned by the lack of women in the sample. However, since CK levels in men are usually higher than in women [52,53,54], we preferred to study a more homogeneous and representative sample. Nevertheless, our study had an important strength because there are no studies that have analyzed the agreement between quantitative measurements of plasmatic and salivary DC, TC, SB, and CK as biomarkers capable of providing a reliable indication of exercise-induced oxidative stress and muscle damage in athletes. Further studies examining whether the levels of salivary DC, TC, SB, and CK can reliably reflect plasma concentrations in male and female athletes who represent different sports will be important.

## 5. Conclusions

This study demonstrates that specific HICE for rowers and HIIEs for runners and swimmers induce lipid peroxidation chain reactions and muscle damage, which can be identified by the measurements of DC, TC, SB, and CK in both blood plasma and saliva. An important finding in that original study was that the concentrations of salivary DC, TC, SB, and CK are able to represent plasma levels in athletes under exercise conditions. Visual examination of the Bland–Altman plots allowed us to detect the high agreement between quantitative measurements of plasmatic and salivary DC, TC, SB, and CK both at rest and following exercise in each athlete group. The established relationship between plasmatic and salivary DC, TC, SB, and CK makes it possible to consider the use of saliva as a similarly reliable alternative to blood analysis in athletes for monitoring oxidative stress and muscle damage imposed by the exercise.

## Figures and Tables

**Table 1:** Baseline characteristics of athletes.

	Runners	Swimmers	Rowers
Age (years)	19.7 ± 1.2	19.4 ± 1.0	19.6 ± 1.0
Height (cm)	173.2 ± 3.1	173.7 ± 1.3	173.0 ± 3.0
Body mass (kg)	63.4 ± 1.7	64.6 ± 2.4	63.0 ± 2.3
BMI (kg/m^2^)	21.2 ± 0.5	21.4 ± 0.6	21.0 ± 0.6

Values are mean ± SD.

**Table 2:** Exercise performance of athletes.

	HIIE (Runners)	HIIE (Swimmers)	HICE (Rowers)
Time to completion (s)	11.15 ± 0.14	-	-
FINA points (a.u.)	-	621.00 ± 34.23	-
Time to completion (min)	-	-	6.36 ± 0.04

Values are mean ± SD. HIIE: high-intensity interval exercise; HICE: high-intensity continuous exercise.

## Data Availability

All relevant data analyzed during the current trial are included in the article. Access to raw datasets may be provided upon reasonable request to the corresponding author.
